# CgCFEM1 and CgCFEM2 modulate virulence in *Colletotrichum gloeosporioides* by integrated regulation of TOR and cAMP-PKA signaling pathways

**DOI:** 10.1186/s12866-026-04969-x

**Published:** 2026-04-28

**Authors:** Liping Feng, Wenjie Liu, Qiannan Wang, Bang An, Hongli Luo

**Affiliations:** 1 National Key Laboratory for Tropical Crop Breeding, School of Breeding and Multiplication (Sanya Institute of Breeding and Multiplication), School of Tropical Agriculture and Forestry (School of Agriculture and Rural Affairs and School of Rural Revitalization), Hainan University, Sanya, 572025 China; 2 Key Laboratory of Agro-Forestry Environmental Processes and Ecological Regulation of Hainan Province, School of Ecology, Hainan University, Haikou, 570228 China

**Keywords:** *Colletotrichum gloeosporioides*, Effector, CgCFEM2, Appressoria, TOR, CAMP-PKA

## Abstract

**Background:**

*Colletotrichum gloeosporioides* causes anthracnose in multiple plants via specialized infection structures, which are regulated by conserved pathways including the Target of rapamycin (TOR) and cyclic adenosine monophosphate-protein kinase A (cAMP-PKA). Effectors with Common in Fungal Extracellular Membranes (CFEM) domain are critical virulence factors. Although CgCFEM1 has been characterized, the role of its closest homolog CgCFEM2 and their potential interplay remains unknown.

**Results:**

This study demonstrates that CgCFEM2 is a crucial pathogenicity effector, with its gene expression highly induced during infection. Pathogenicity assays on rubber tree leaves revealed that the Δ*CgCFEM2* mutant was severely attenuated in virulence, and the double mutant Δ*CgCFEM1/2* exhibited an even more severe defect, indicating CgCFEM1 and CgCFEM2 play complementary yet distinct roles in pathogenicity. Both effectors were essential for normal conidia morphology, conidiation, and the critical transition from appressoria to invasive hyphae. Mechanistically, we discovered that loss of CgCFEM1 and CgCFEM2 leads to deregulated cell cycle progression during infection-related development, resulting in aberrant germ tube elongation and impaired appressorium formation. Additionally, the mutants exhibited hyperactive phosphorylation of p70-S6 kinase (p70 S6K) and insensitivity to rapamycin, indicating the involvement of TOR signaling. A key finding is that both CgCFEM1 and CgCFEM2 positively regulate intracellular cAMP levels, but through fundamentally distinct targets: CgCFEM1 promotes cAMP synthesis by upregulating the adenylate cyclase gene *CgMac1*, whereas CgCFEM2 inhibits cAMP degradation by repressing the phosphodiesterase gene *CgPdeH*.​ Consequently, the loss of these effectors led to reduced cAMP levels and impaired phosphorylation of MAP kinase Pmk1.

**Conclusions:**

Our findings establish that CgCFEM1 and CgCFEM2 as pivotal, synergistic regulators of the *C. gloeosporioides* infection cycle. They converge on elevating cAMP levels via different mechanisms: synthesis versus degradation. They also influence TOR signaling. Together, they ensure proper fungal development and pathogenicity. This study provides novel insights into the sophisticated effector network employed by phytopathogenic fungi to achieve successful infection.

**Supplementary Information:**

The online version contains supplementary material available at 10.1186/s12866-026-04969-x.

## Background

The ascomycete fungus *Colletotrichum gloeosporioides* is a pervasive phytopathogen responsible for anthracnose diseases in a multitude of economically vital host plants, including the rubber tree (*Hevea brasiliensis*) [1, 2]. The successful colonization by *C. gloeosporioides* hinges on a meticulously orchestrated infection cascade [3]. This process begins with the attachment of conidia to the host surface, followed by the formation of specialized infection structure called appressoria [4]. The appressoria generate enormous internal turgor pressure, enabling the direct breach of the host cuticle. Following penetration, the fungus develops invasive hyphae to proliferate within the host tissue, necessitating the suppression of plant immune responses for successful infection [5].

​​Multiple conserved signaling pathways regulate the morphogenesis of these infection-related structures [6].​ Surface cues activate the cyclic adenosine monophosphate-protein kinase A (cAMP-PKA) signaling pathway, which in turn regulates the formation of functional appressoria [7, 8]. The opposing activities of adenylate cyclase (MAC1) and phosphodiesterase (PDE) collectively regulate intracellular cAMP levels [9]. ​​In *Colletotrichum scovillei*, the key cAMP signaling components (CsAc1/CsCap1/CsPdeH) regulate key stages of fungal development​ (including development of vegetative mycelia, conidia, and appressoria) by controlling intracellular cAMP levels and subsequent signaling events [8]. ​​

The Pmk1-mitogen-activated protein kinase (MAPK) signaling pathway serves as an additional master regulator of infection-related development [6]. ​​​​In *Pyricularia oryzae*, the kelch-BTB domain protein PoRal2 functions as a critical nexus that integrates the cAMP-PKA and Pmk1 MAPK signaling pathways to regulate appressorium morphogenesis. Consequently, knockout of *PoRAL2* results in defective signal integration, reduced Pmk1 phosphorylation, and loss of responsiveness to exogenous cAMP [10].

The target of rapamycin (TOR) signaling pathway also contributes to appressorium function [11–13]. This pathway integrates nutrient and stress signals to control cell growth and development. In *Magnaporthe oryzae*, the MoWhi2-MoPsr1 complex is essential for precise appressorium formation and full pathogenicity [14]. This complex functions by negatively regulating the MoTOR signaling pathway. Its disruption leads to inappropriate TOR activation, aberrant cell cycle progression, and the formation of multiple appressoria per conidium [14]. MoTOR signaling is also involved in coordinating lipid metabolism, exemplified by its control of Nem1-mediated lipid homeostasis during conidium and appressorium formation [15].​ ​​This central role of TOR signaling in infection structure development is further underscored in the necrotrophic fungus *Sclerotinia sclerotiorum*, where silencing *SsTOR* severely disrupts the formation of functional appressoria and abolishes pathogenicity [13].​

To manipulate host defenses and establish compatibility, fungal pathogens deploy a plethora of effectors. A particularly intriguing class of effectors contains the Common in Fungal Extracellular Membranes (CFEM) domain, a motif unique to fungi. CFEM proteins, which can be surface-localized or secreted, have been implicated in diverse functions including immune evasion, iron acquisition and pathogenicity [16–22]. These functions have been characterized in various fungal systems, including the phytopathogen *Botrytis cinerea*, *Fusarium graminearum*,* M. oryzae*, *Lasiodiplodia theobromae*, *Setosphaeria turcica*, as well as the human pathogen *Aspergillus fumigatus* and *Candida albicans* [16–22]. Our previous work identified CgCFEM1 as a critical virulence factor in *C. gloeosporioides*, demonstrating its role in regulating TOR-mediated conidial and appressorial morphogenesis and in suppressing the host defense responses [23].

The genome of *C. gloeosporioides* encodes a repertoire of CFEM effectors, yet the biological functions of the majority remain unclear. Phylogenetic analysis reveals that CgCFEM2 is the closest homolog to the characterized CgCFEM1, suggesting a potential similar or complementary role in pathogenesis. This study presents a comprehensive functional characterization of CgCFEM2 and its interplay with CgCFEM1. We employed gene knockout and double-mutant strategies to dissect their collective and individual contributions to pathogenicity. We investigated their roles in stress tolerance, conidiation, appressoria formation, and invasive hyphae development. Furthermore, we investigated the molecular mechanisms, providing evidence that CgCFEM1 and CgCFEM2 converge on critical signaling hubs, positively regulating cAMP levels—through distinct molecular targets—and modulating TOR activity to ensure proper cell cycle progression and the differentiation of functional infection structures. Our findings establish that CgCFEM1 and CgCFEM2 are key factors that synergistically orchestrate the infection cycle of *C. gloeosporioides* by integrating into central developmental and signaling pathways.

## Methods

### Fungal and plants materials, and growth conditions

#### Fungal material and culture conditions

The wild type (WT) strain of *C. gloeosporioides* (BioSample: SAMN17266943, obtained originally from *Hevea brasiliensis*) was used. Fungal material was maintained on potato dextrose agar (PDA) plates and incubated at 28 °C in darkness.

#### Plant material and growth conditions

Seedlings of the *Hevea brasiliensis* (cultivar 'Reyan 7–33–97') were purchased from the National Rubber Tree Germplasm Repository (Danzhou, Hainan, China). As commercially cultivated plants, no voucher specimen was deposited. All seedlings were grown in a plant growth room maintained at 28 °C.

### Gene expression analysis

Following a published method, the procedures of gene expression analysis, encompassing total RNA extraction, cDNA synthesis, and quantitative RT-PCR (qRT-PCR) were performed [23]. Briefly, total RNA was extracted from fungal samples (mycelia, conidia, appressoria) and from inoculated rubber tree leaves collected at various time points. The determination of gene expression was then calculated by the 2 ^−ΔΔCt^ method from three biological and four technical replicates, with the *CgActin* gene serving as the endogenous control.

### Construction of mutant strains

To generate the *CgCFEM2* knockout mutant (Δ*CgCFEM2*), a split-marker recombination strategy was employed in the WT strain (Figure S2A), following a previously established method [24]. Putative transformants were screened by PCR, purified to homokaryons, and the full-length *CgCFEM2* sequence was amplified from the knockout strains, with the WT strain serving as a positive control (Figure S2C). To confirm the *CgCFEM*2 deletion, Southern blot analysis was performed. Genomic DNA (20 μg) from WT and Δ*CgCFEM*2 strains was digested with *Spe*I, fractionated on a 0.8% agarose gel, and transferred to a nylon membrane. A digoxigenin-labeled probe specific to the upstream flanking region of *CgCFEM*2 was used for hybridization. Subsequently, the Δ*CgCFEM2* strain was used to create a *CgCFEM1/CgCFEM2* double mutant (Δ*CgCFEM1/2*) via protoplast transformation (Figure S3). For complementation (Figure S2B), the Δ*CgCFEM2* strain was transformed with an expression cassette containing the full-length *CgCFEM2* gene driven by its native promoter and the selectable marker gene *HPT* (hygromycin phosphotransferase).

### Evaluation of pathogenicity

To assess pathogenicity, fresh leaves of the rubber tree seedlings were collected and subjected to wounding or left intact before inoculation with 5 µL droplets of a conidial suspension (2 × 10^5^ conidia mL⁻^1^) as previously described [24]. Disease progression was evaluated at 4 days post-inoculation (dpi). Each treatment included three biological replicates (10 leaves per replicate), and the entire assay was independently performed three times.

### Assessment of fungal growth and conidiation

Fungal growth​​ was assessed by inoculating a 5-mm-diameter hyphal disk taken from the actively growing colony edge onto PDA medium. After 4 days of incubation, the colony diameter was measured, and the morphology was documented. ​​To evaluate conidiation​​, 1 mL conidial suspensions (containing 1 × 10^3^ conidia) of *C. gloeosporioides* strains were added into a fixed volume (50 mL) of liquid complete medium (CM). After incubation with shaking at 120 rpm for 3 days, the conidia production was quantified using a microscope. Liquid shake culture was employed as it allows for quantitative and reproducible comparisons of conidial yield under uniform conditions, minimizing variability inherent to solid media [25]. All experiments were performed with four replicates per sample and repeated three times independently. For each replicate, ten random microscope fields were examined.

### Assessment of stress tolerance

To assess stress tolerance, a 5-mm-diameter hyphal disk of different *C. gloeosporioides* strains were transferred onto minimal medium (MM) plates amended with cell wall‐perturbing agents [including CFW (200 mg L⁻^1^), Congo red (0.35 mg L⁻^1^), and SDS (0.005%, wt/vol)] and the TOR inhibitor rapamycin (Rap, 50 nM). After 4 days of incubation, the colony diameter was measured, and the morphology was documented. Untreated cultures grown under the same conditions served as the control. The experiment included three replicates per treatment and was repeated twice independently.

### Y2H assay

The yeast two-hybrid (Y2H) assay was performed as described [26]. Recombinant plasmids pGBKT7-CgCFEM1 (BD-CgCFEM1) and pGADT7-CgCFEM2 (AD-CgCFEM2) were co-transformed into the *Saccharomyces cerevisiae* Y2HGold competent cell (Weidi, China). Transformants were selected on DDO plates and further screened on QDO plates supplemented with X-α-Gal (20 μg mL^−1^). The positive (AD-T + BD-53) and negative (AD-T + BD- Lam) control pairs were processed in parallel.

### Assessment of appressorium formation and penetration ability

The indicated strains were evaluated for appressorium formation on polystyrene plates and for penetration ability on onion epidermis, following a described method [23] with modifications. Briefly, for appressorium development, droplets of conidial suspension (3 × 10^5^ conidia mL⁻^1^) were inoculated onto polystyrene plates. Formation was quantified at 12- and 24 h post-inoculation (hpi) using a Leica DM2000 microscope, with at least 100 conidia counted per replicate. To test the involvement of CgCFEM1 and CgCFEM2 in the TOR signaling pathway, conidial suspensions were treated with 200 nM Rap and analyzed at 24 hpi. For penetration assays, conidia (2 × 10^5^ conidia mL⁻^1^) were inoculated onto onion epidermis, and infection structures were examined at 24 hpi. All assays were performed with three replicates per treatment (≥ 100 conidia per replicate) and repeated twice independently. Relative formation rates were calculated from three independent experiments.

### Cell staining

To visualize septa, conidial suspensions (1 × 10^5^ conidia mL⁻^1^) were dropped onto polystyrene plates for approximately 45 min and then stained with CFW for 10 min before fluorescence microscopy under UV illumination. For nuclear staining, conidia were treated with 5 μg mL⁻1 2-(4-Amidinophenyl)−6-indolecarbamidine dihydrochloride (DAPI) for 3–5 min and observed under the same conditions. Similarly, conidial suspensions were dropped onto polystyrene plates and incubated for approximately 6–8 h to induce germ tube formation, ​​and were then stained​​ following the previously described method [23]. All experiments were performed with four replicates per sample and repeated three times independently.

### Yeast signal peptide trap assay

To determine whether CgCFEM2 possesses a functional signal peptide, the predicted N-terminal signal peptide sequence was cloned into the pSUC2 vector and transformed into YTK12 yeast strain. Transformants were spotted onto YPR agar plates containing sucrose as the sole carbon source. The empty pSUC2 vector and Avr1b signal peptide served as negative and positive controls, respectively. Detailed results are presented in Figure S4.

### Reactive oxygen species burst assay in rubber tree mesophyll protoplasm

ROS levels in rubber tree mesophyll protoplasm expressing CgCFEM2-Flag or empty vector were measured upon chitin treatment (200 μg mL⁻^1^) as described [23]. Detailed results are presented in Figure S4.

### Immunoblot

To assess the phosphorylation status of S6K1, mycelia from WT and mutant strains were harvested after 8 h Rap (200 nM) treatment (mycelia without treatment served as the control) and the total protein was extracted from equal amounts of the mycelial powder using a freshly prepared cell lysis buffer following a described method [23]. Phosphorylation of S6K1 was detected with antibody against phospho-p70 S6K (Proteintech, China), p70 S6K (Proteintech, China) and GAPDH (Proteintech, China).

For detecting Pmk1 activity, antibody against phospho-p42/44 (Proteintech, China), p42/44 (Proteintech, China) and GAPDH was used.

### Quantification of cAMP levels

Measurement of intracellular cAMP levels was performed as previously described [24]. Briefly, appressoria induced on polystyrene plates for 24 h were harvested using a cell scraper. Appressorial samples (0.1 g) from each strain were homogenized in 1 mL HCl (0.1 M). The homogenates were vortexed thoroughly and then centrifuged for 10 min at a lower speed (800 × g). The resulting supernatant was subjected to a commercial cAMP ELISA kit (Shanghai Guyan Industrial, China) to determine the cAMP concentration.

### Statistical analysis

All data were analyzed using SPSS Statistics (IBM). One-way ANOVA, followed by Duncan's test for mean comparisons, was applied to single-factor experiments, with a significance threshold of *p* < 0.05.

## Results

### CgCFEM2 contributed to pathogenicity

The genome of *C. gloeosporioides* encodes five members of CFEM effectors (Table S1; named as CgCFEM1-5). Previous work identified CgCFEM1 as a key virulence factor that regulates TOR-mediated conidial and appressorial morphogenesis while suppressing host defense responses in rubber tree [23]. A phylogenetic analysis of the CFEM domains was conducted to explore functional relationships among these CgCFEM effectors (Figure S1A). The evolutionary tree results showed that CgCFEM2 was closer to CgCFEM1 with sequence identities in the CFEM domain of 31% (Figure S1B), which may be another candidate of key effectors in *C. gloeosporioides* to be further studied.

**Fig. 1:**
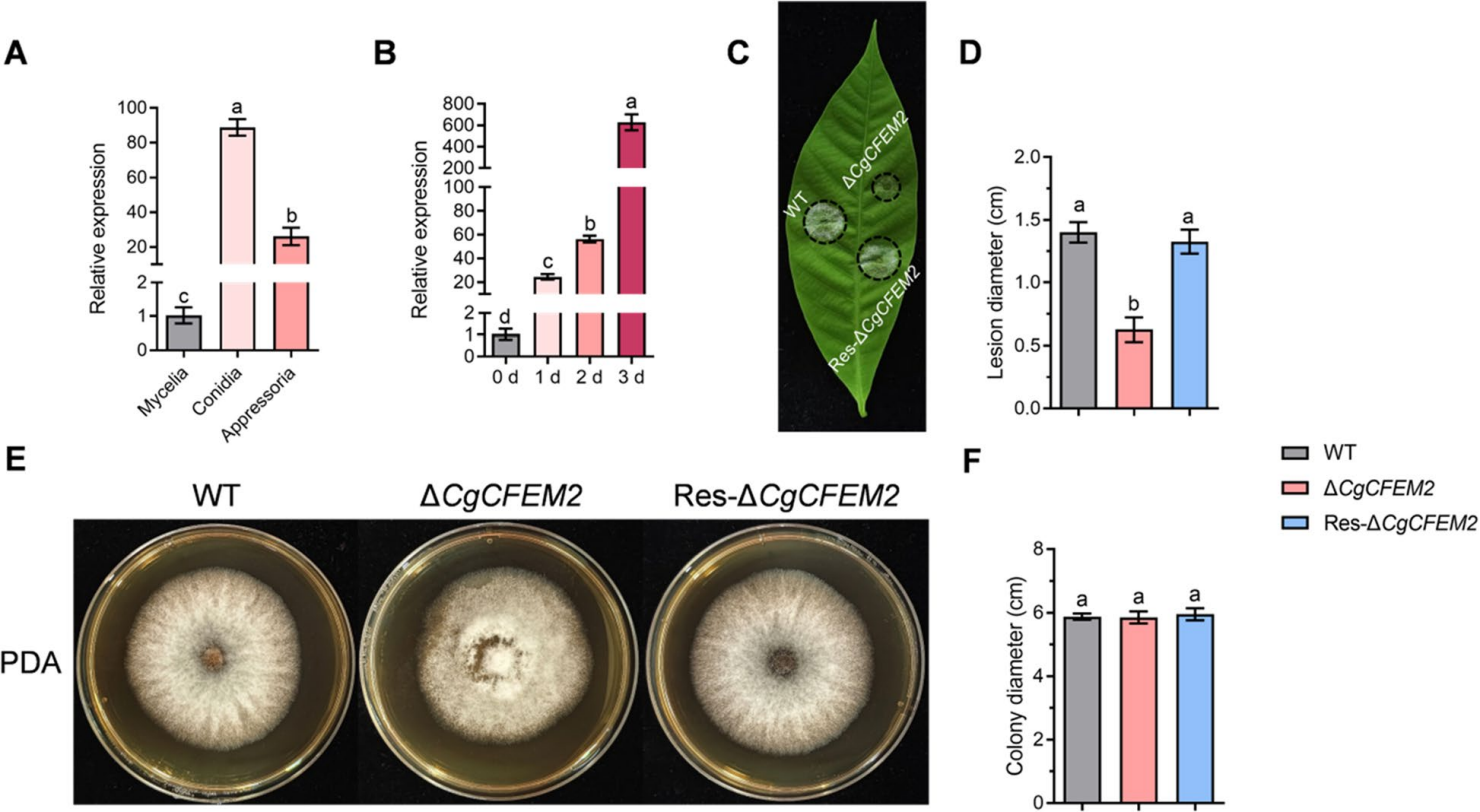
CgCFEM2 regulates virulence in *C. gloeosporioides*. **A** Expression profile of *CgCFEM2* across distinct developmental phases. **B** Expression profile of *CgCFEM2* at various time points post-inoculation. **C** Pathogenicity assay of the indicated strains on detached rubber tree leaves. Representative image show disease symptoms. **D** Quantitative analysis of lesion diameter shown in (**C**). **E** Colony morphology of the indicated strains on PDA plates 4 dpi. **F** Quantitative analysis of the colony diameters shown in (**E**). Data represent the mean ± SD in three independent experimental repeats, and different letters above the columns in (**D**) and (**F**) indicate statistically significant differences (*p* < 0.05)

We first examined the expression pattern of CgCFEM2 during fungal development and host infection using qRT‑PCR. The results (Fig. 1A and B) showed that *CgCFEM2* transcripts were induced significantly in conidia by around 80-fold relative to mycelia and 4 times more than that in appressoria. Besides, on inoculated rubber tree leaves, the expression of *CgCFEM2* was induced along with the inoculation time and reached a peak at 3 dpi by around 600-fold up-regulation. These results imply a potential role for CgCFEM2 in conidiation and virulence.

To further investigate its function, we generated and verified *CgCFEM2* knockout mutant (Δ*CgCFEM2*) and complemented strain (Res-Δ*CgCFEM2*) by PCR and Southern blot analysis (Figure S2C and S2D). After that, we analysed the virulence of the indicated strains, including WT, ∆*CgCFEM2* and Res-∆*CgCFEM2* strains, on detached pre-wounded rubber tree leaves. Pathogenicity tests results showed that all of them produced anthracnose lesions at 4 dpi (Fig. 1C). However, the necrotic lesions caused by Δ*CgCFEM2* were significantly smaller than those incited by the WT and complemented strains statistically (Fig. 1D). In contrast, Δ*CgCFEM2* showed no obvious defect in vegetative growth on PDA medium (Fig. 1E and F). These results strongly support the view that CgCFEM2 plays an infection-specific role in virulence of *C. gloeosporioides*.

### CgCFEM2 is required for tolerance to cell wall stressors

**Fig. 2:**
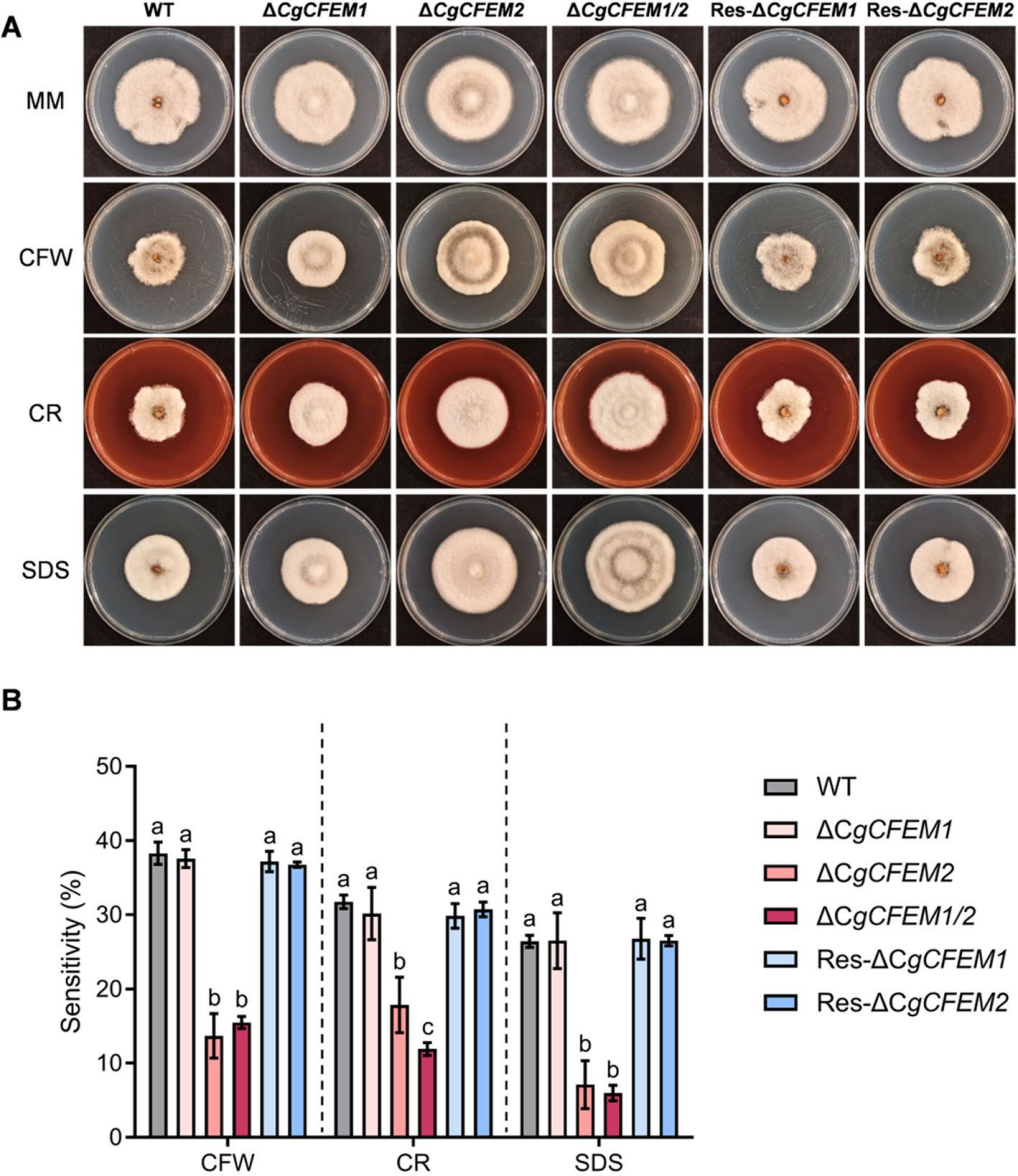
CgCFEM2 is specifically required for tolerance to CFW and SDS. **A** Colony morphology of the indicated strains on chemical-amended MM plates at 4 dpi. **B** Sensitivity quantification under various chemicals shown in (**A**). Data represent the mean ± SD in three independent experimental repeats, and different letters above the columns in (**B**) indicate statistically significant differences (*p* < 0.05)

To further explore the relevance of *CgCFEM1* and *CgCFEM2*, double-mutant (named as Δ*CgCFEM1/2*) was generated (Figure S3). Furthermore, the role of these two CFEM effectors in stress tolerance was investigated. Fungal strains were cultured on MM amended with various chemicals. Notably, only the Δ*CgCFEM2* and Δ*CgCFEM1/2* mutants exhibited significantly reduced sensitivity to CFW, CR and SDS, whereas the Δ*CgCFEM1* mutant showed a similar response to the WT and complementation strains (Fig. 2). These results demonstrate that CgCFEM2, but not CgCFEM1, is required for tolerance to these cell wall stressors.

### CgCFEM1 and CgCFEM2 are required for full virulence of *C. gloeosporioides* but do not interact directly

**Fig. 3:**
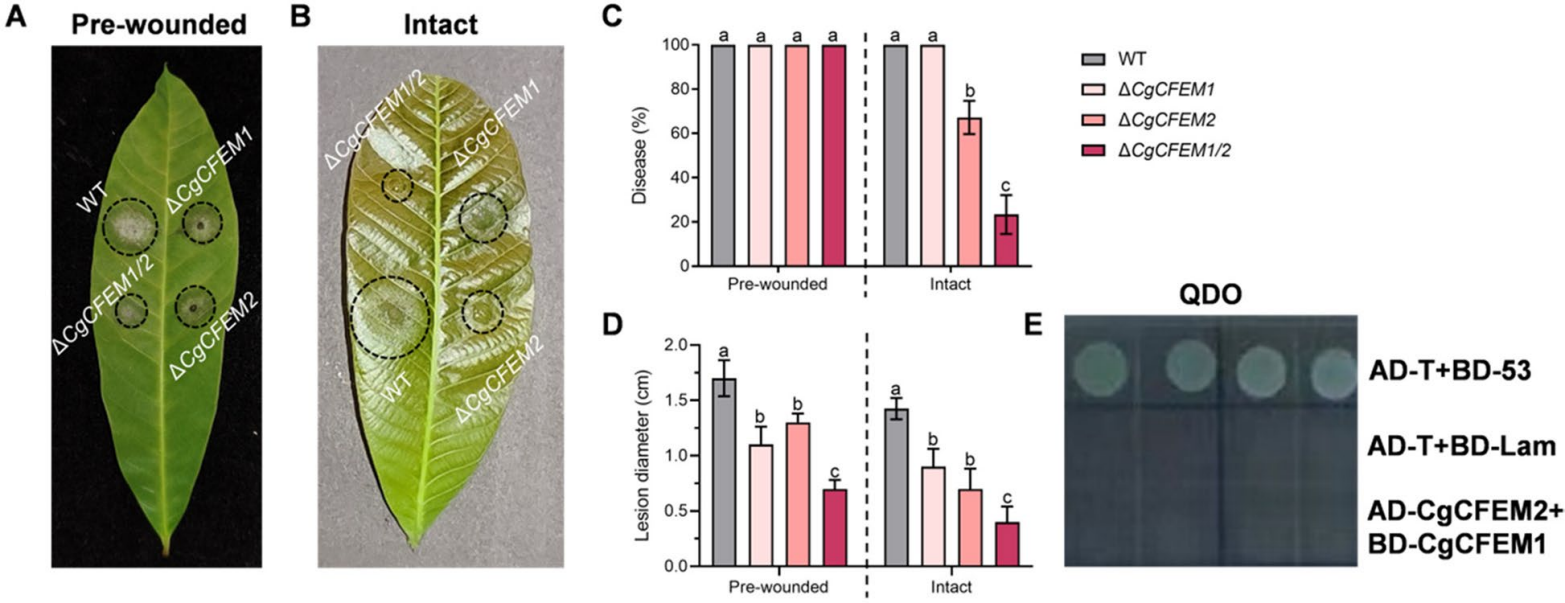
Pathogenicity assay and interaction analysis. **A**, **B** Representative disease symptoms of WT and mutants on pre-wounded (**A**) and intact (**B**) leaves at 4 dpi. **C** Disease incidence of infections shown in (**A**) and (**B**). **D** Quantitative analysis of lesion diameter shown in (**A**) and (**B**). **E** Analysis of protein–protein interaction between CgCFEM1 and CgCFEM2 by Y2H assay. Data represent the mean ± SD in three independent experimental repeats, and different letters above the columns in (**C**) and (**D**) indicate statistically significant differences (*p* < 0.05)

To elucidate the individual and combined roles of CgCFEM1 and CgCFEM2 in pathogenesis, we performed infection assays on both pre-wounded and intact rubber tree leaves. On pre-wounded leaves, all strains caused typical anthracnose lesions with 100% disease incidence (Fig. 3A and C). In contrast, on intact leaves, the disease incidence for Δ*CgCFEM2* and Δ*CgCFEM1/2 *dropped to 67% and 23%, respectively (Fig. 3C). Furthermore, all mutants produced significantly smaller lesions than those of the WT on both leaf types (Fig. 3A, B, D). Notably, the double mutant Δ*CgCFEM1/2* was significantly more attenuated in virulence than either single mutant, indicating additive effects on disease development. A Y2H assay between CgCFEM1 and CgCFEM2 showed no interaction (Fig. 3E), suggesting that their synergistic contribution to virulence is not mediated by direct physical binding. Collectively, these findings demonstrate that CgCFEM1 and CgCFEM2 are pivotal for full virulence, functioning through non-interacting pathways.

### CgCFEM1 and CgCFEM2 are required for conidial morphogenesis and conidiation in *C. gloeosporioides*

**Fig. 4:**
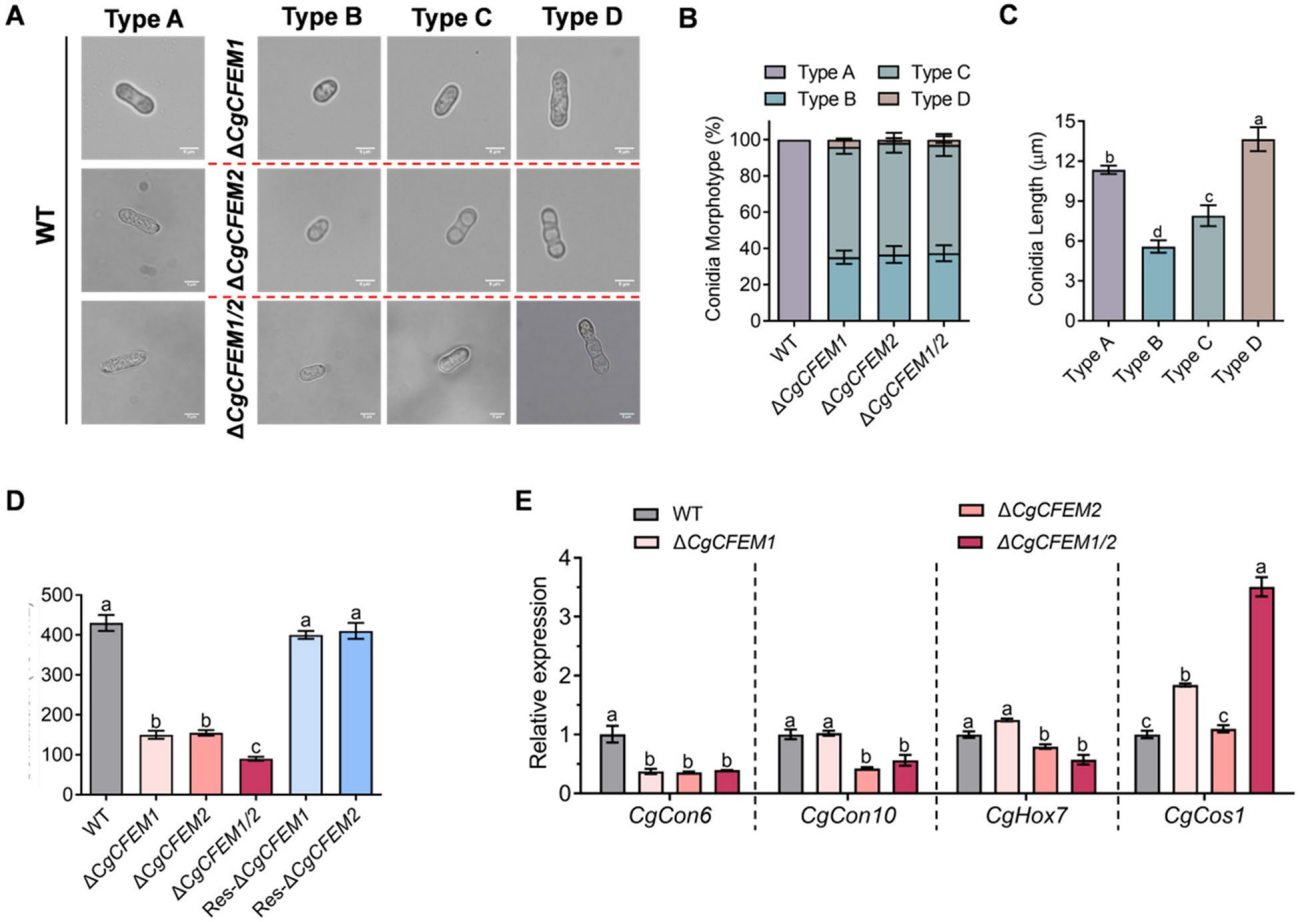
Effects of CgCFEM1 and CgCFEM2 deletion on conidial development in *C. gloeosporioides*. **A** Morphology of conidia from WT and mutant strains. Conidia were classified into four types: Type A (normal), Type B (near-spherical), Type C (shorter than normal), and Type D (longer than normal). Scale Bars = 5 µm. **B** Frequency distribution of conidial types shown in (**A**). **C** Length measurements of the different conidial types. **D** Conidiation of the indicated strains at 3 dpi. **E** Expression levels of conidiation-related genes in WT and mutant strains. Data represent the mean ± SD in three independent experimental repeats, and different letters above the columns in (**C**), (**D**) and (**E**) indicate statistically significant differences (*p* < 0.05)

To explore the basis for the reduced virulence in Δ*CgCFEM2* and Δ*CgCFEM1/2* mutants, we analysed their conidial development. While conidial germination was unaffected in all mutants compared to the WT (Figure S5), both Δ*CgCFEM2* and the double mutant Δ*CgCFEM1/2* exhibited abnormal conidial morphology, similar to the previously reported Δ*CgCFEM1* mutant [23]. The mutant conidia displayed substantial morphological diversity, including near-spherical (Type B, ~ 36%), shorter (Type C, ~ 61%), and elongated (Type D, ~ 3%) forms, contrasting with the uniform WT conidia (Type A, 100%) (Fig. 4A–C). Furthermore, conidiation was severely impaired in all mutants, with yields reduced to 35% (Δ*CgCFEM1*), 36% (Δ*CgCFEM2*), and 21% (Δ*CgCFEM1/2*) of WT levels (Fig. 4D). Expression analysis of key conidiation-related genes revealed downregulation of *CgCon6*, *CgCon10*, and *CgHox7* in Δ*CgCFEM2* and Δ*CgCFEM1/2*, and upregulation of *CgCos1* in Δ*CgCFEM1* and Δ*CgCFEM1/2* (Fig. 4E). These results demonstrate that both CgCFEM1 and CgCFEM2 are essential for normal conidial morphogenesis and conidiation.

### CgCFEM1 and CgCFEM2 are essential for the development of infection structures in *C. gloeosporioides*

**Fig. 5:**
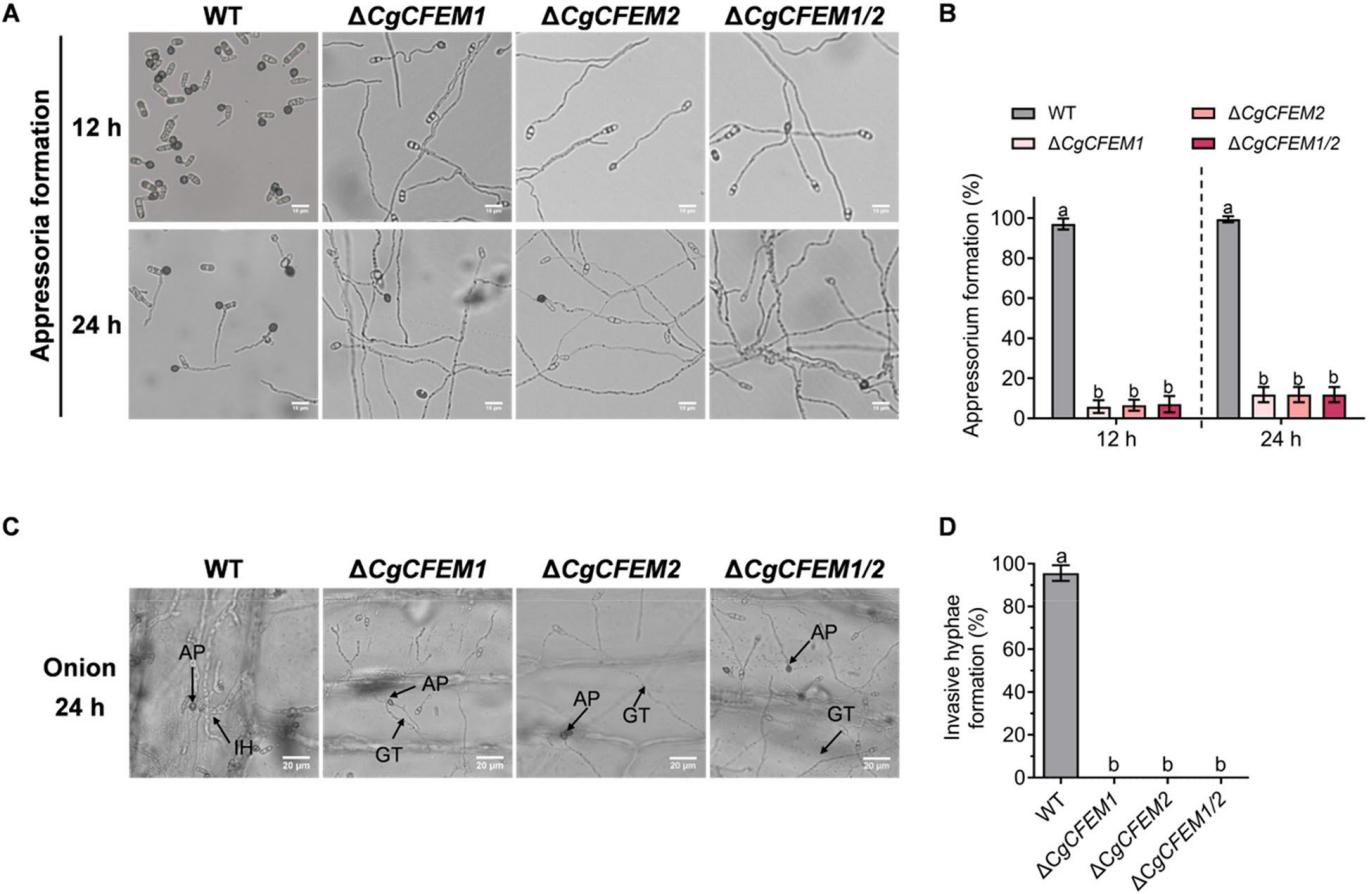
Analysis of invasive structure development. **A** Appressoria produced by WT and mutant strains on hydrophobic polystyrene plates at 12 and 24 hpi. Scale bars = 10 μm. **B** Quantification of appressorium formation rates shown in (**A**). **C** Appressorium development and penetration on onion epidermis at 24 hpi. GT, germ tube; AP, appressorium; IH, invasive hypha. Scale bars = 20 μm. **D** Penetration efficiency, measured as the percentage of appressoria that formed invasive hyphae, as shown in (**C**). Data represent the mean ± SD in three independent experimental repeats, and different letters above the columns in (**B**) and (**D**) indicate statistically significant differences (*p* < 0.05)

To determine the basis for the attenuated virulence in *CgCFEM* mutants, we assessed their ability to form infection structures. On artificial hydrophobic surfaces (polystyrene plates), the Δ*CgCFEM1*, Δ*CgCFEM2*, and Δ*CgCFEM1/2* mutants all exhibited significantly reduced rates of appressorium formation compared to WT (Fig. 5A and B). Furthermore, penetration assays on onion epidermis revealed that while over 90% of WT appressoria gave rise to invasive hyphae within 24 h, all three mutants were severely impaired, producing only a few abnormal appressoria and virtually no invasive hyphae (Fig. 5C and D). This pronounced defect at 24 hpi indicates that the mutants are compromised in the timely development of functional invasive hyphae, consistent with their reduced virulence on rubber tree leaves (Fig. 3). These results demonstrate that both CgCFEM1 and CgCFEM2 are critical for efficient host penetration and colonization in *C. gloeosporioides*.

### CgCFEM1 and CgCFEM2 are involved in cell cycle progression in conidia and germ tubes

**Fig. 6:**
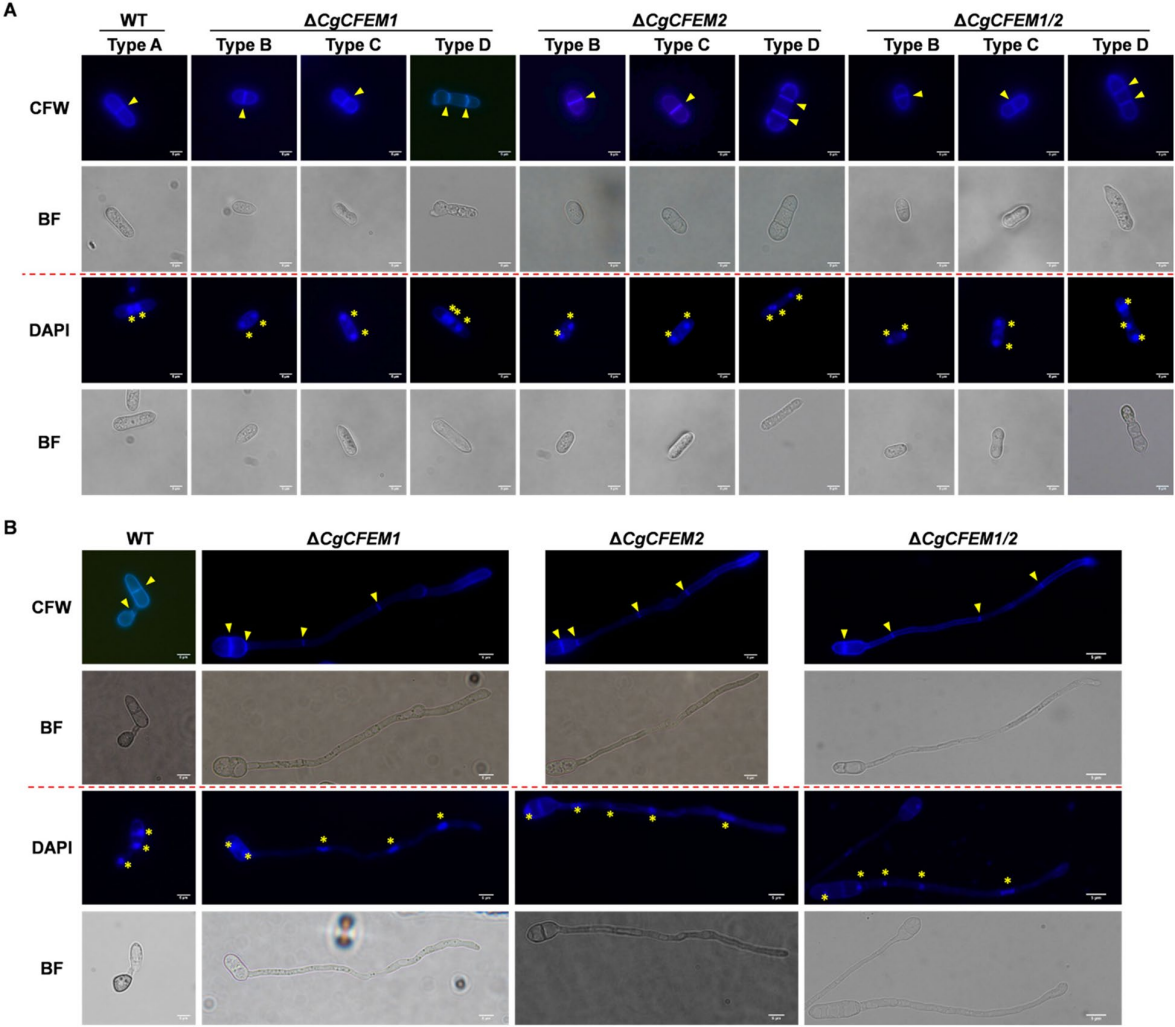
Septal​ and nuclear staining of conidia and germ tubes in WT and mutant strains. **A** Staining of conidia from WT and mutant strains with CFW (septal staining) and DAPI (nuclear staining). **B** Staining of germ tubes from the indicated strains at 8 hpi. Yellow triangles indicate septa, and yellow asterisks mark nuclei. Scale Bars = 5 µm. Quantitative analysis of conidial morphology and length distribution for each strain is presented in Fig. 4B and C

As shown in Fig. 4A-C, the conidial length of all three mutants differed significantly from that of the WT strain. To determine whether these changes in conidial length resulted from altered cell size or nuclear division, we performed CFW and DAPI staining. The results revealed that Type B and C conidia from all mutants, as well as Type A conidia from the WT strain, contained one septum and two nuclei. In contrast, Type D conidia from all mutants possessed two septa and three nuclei (Fig. 6A). Furthermore, unlike the WT, approximately 90% of the mutant conidia failed to produce appressoria and instead developed elongated germ tubes (Fig. 5A and B). To investigate the underlying reason for these defects, we induced appressorium formation by incubating conidial suspensions on polystyrene plates for 6–8 h, followed by CFW and DAPI staining. The staining showed that WT conidia formed normal appressoria and contained two septa and three nuclei. Conversely, most mutant conidia exhibited four septa and five nuclei (Fig. 6B). Collectively, these data suggest that the loss of CgCFEM1 and CgCFEM2 leads to excessive mitosis in germ tubes, indicating deregulated cell cycle progression during these developmental stages. Previous studies in *M*. *oryzae* have demonstrated that proper cell cycle control is essential for appressorium differentiation, and that uncontrolled mitosis leads to elongated germ tubes and impaired infection structure formation [27].

### CgCFEM1 and CgCFEM2 regulate appressorium formation via the TOR signaling pathway

**Fig. 7:**
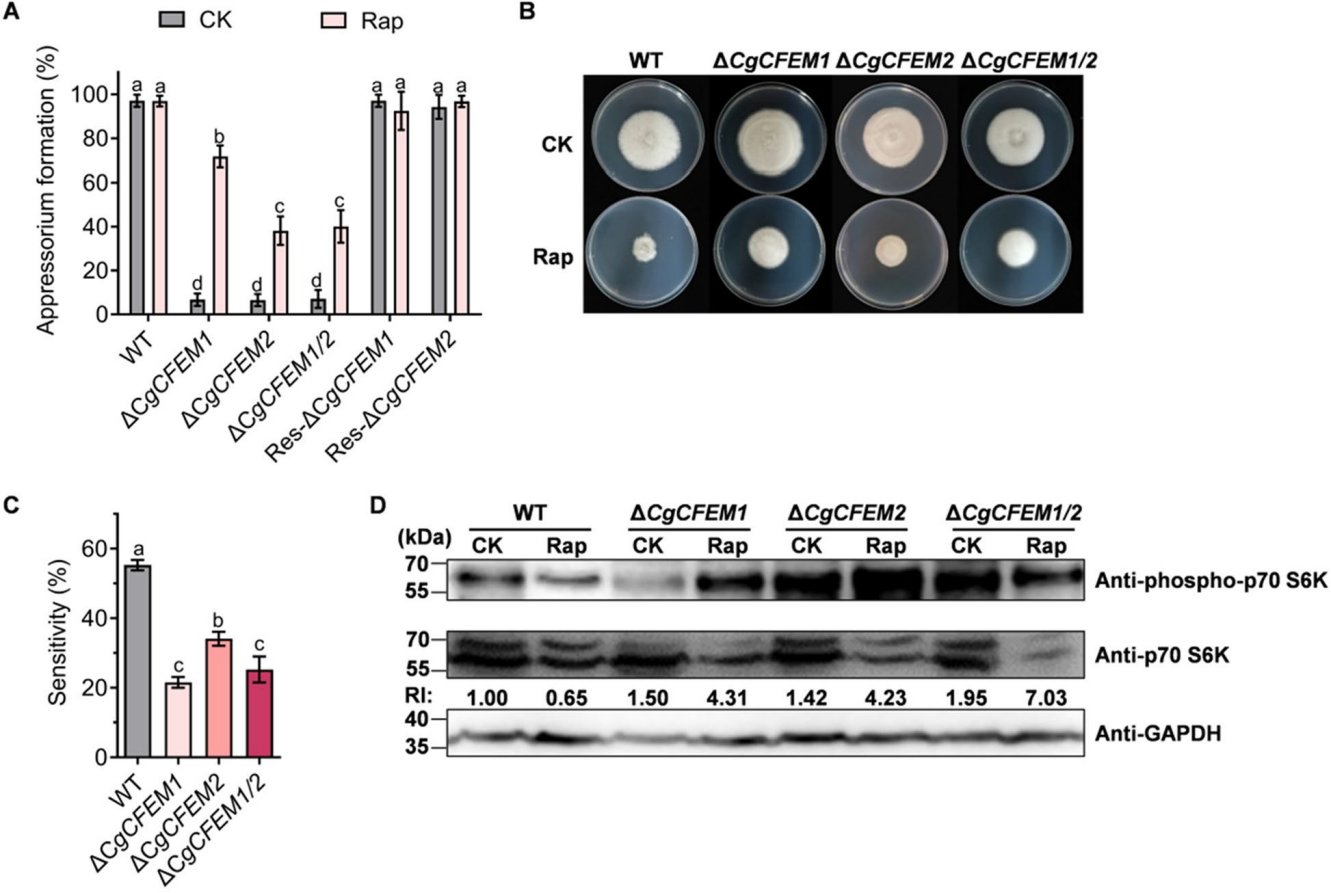
CgCFEM1 and CgCFEM2 impact appressorium formation via the TOR signaling pathway​. **A** Appressorium formation rates of the indicated strains with or without 100 nM rapamycin (Rap) treatment. CK, control. **B **Colony morphology of WT, Δ*CgCFEM1*, Δ*CgCFEM2* and Δ*CgCFEM1/2* strains incubated on MM supplemented with 50 nM Rap at 4 dpi. **C** Rapamycin sensitivity of the strains shown in (**B**), quantified as relative colony area compared to the untreated control (CK). **D** Western blot analysis of phosphorylated p70-S6 kinase (phospho-p70 S6K) and total p70 S6K levels in the indicated strains under untreated and Rap-treated conditions. RI, relative intensity of phosphorylation, calculated by normalizing phospho-p70 S6K band intensity to total p70 S6K band intensity using ImageJ. Full-length, unprocessed blots are provided in Supplementary Information. Data represent the mean ± SD in three independent experimental repeats, and different letters above the columns in (**A**) and (**C**) indicate statistically significant differences (*p* < 0.05)

Given that rapamycin (Rap) partially restored appressorium formation in Δ*CgCFEM1* [23] and Δ*CgCFEM2* exhibited a similar defect (Fig. 5A), we hypothesized that CgCFEM2 is also involved in TOR signaling. Exogenous Rap partially rescued the appressorium formation defect in both Δ*CgCFEM2* and Δ*CgCFEM1/2* mutants (Fig. 7A). Rap tolerance assays further revealed that all three mutants were significantly less sensitive to Rap (50 nM) than WT (Fig. 7B and C). To directly assess TOR activity, we measured the phosphorylation level of p70-S6 kinase (S6K). Western blot analysis showed elevated S6K phosphorylation in all three mutants compared to that of WT, both with and without Rap treatment (Fig. 7D). These results demonstrate that CgCFEM1 and CgCFEM2 are required for normal TOR signaling during appressorium differentiation and formation.

### CgCFEM1 and CgCFEM2 are involved in cAMP-PKA signaling pathway

**Fig. 8:**
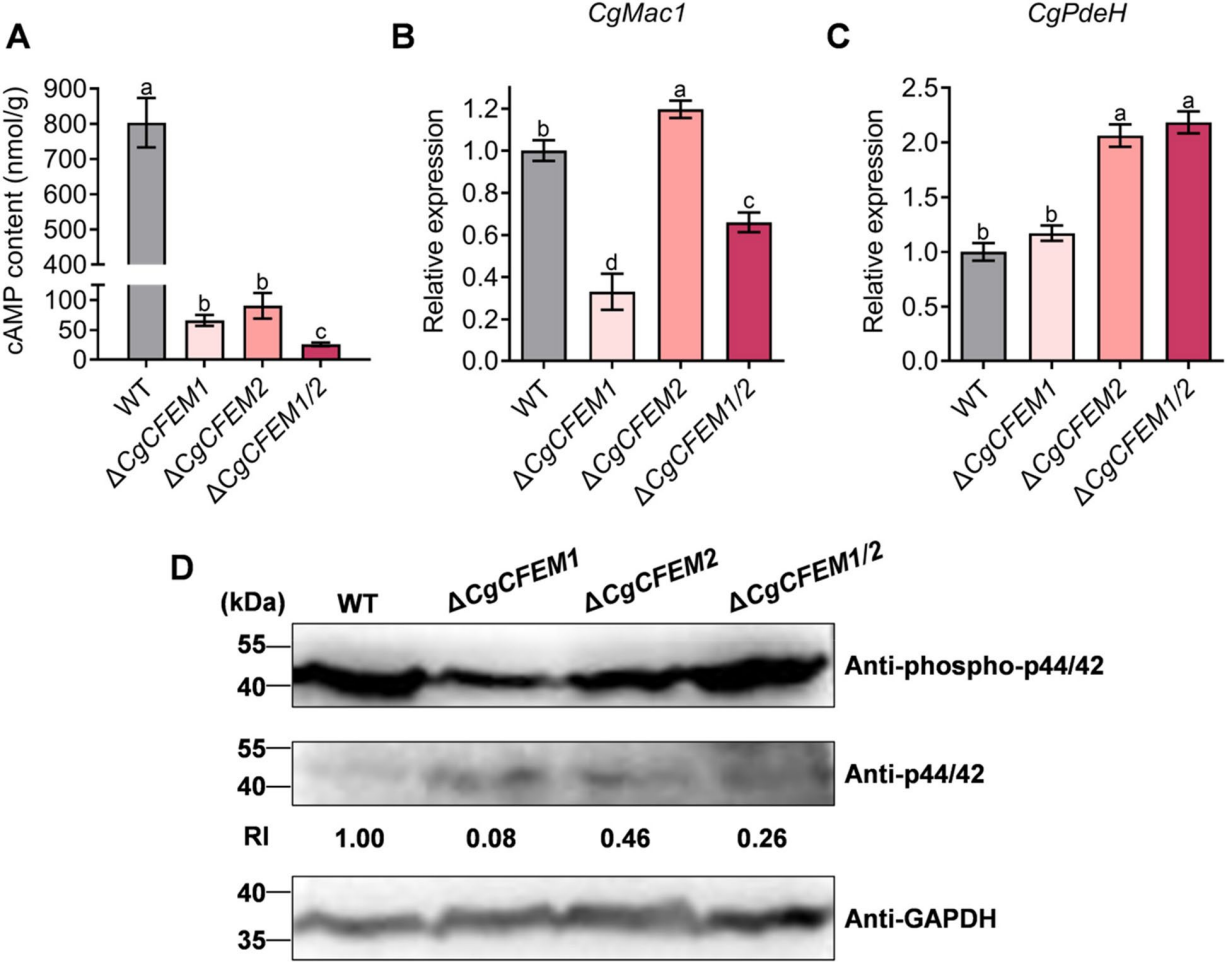
CgCFEM1 and CgCFEM2 regulate intracellular cAMP levels and Pmk1 MAPK activation​. **A**​ Intracellular cAMP concentrations in WT and mutant strains. **B**, **C** Relative expression levels of the cAMP synthesis gene *CgMac1*(B) and the cAMP degradation gene *CgPdeH* (**C**) in the indicated strains. **D** Western blot analysis of Pmk1 phosphorylation in the indicated strains. RI, relative intensity of phosphorylation, calculated by normalizing phospho-Pmk1 band intensity (detected with anti-phospho-p44/42 antibody) to total Pmk1 levels (detected with anti-p44/42 antibody) using ImageJ. Full-length, unprocessed blots are provided in Supplementary Information. Data represent the mean ± SD in three independent experimental repeats, and different letters above the columns in (**A**), (**B**) and (**C**) indicate statistically significant differences (*p* < 0.05)

Given the established role of cAMP-PKA signaling in fungal appressorium development [9], we investigated whether CgCFEM1 and CgCFEM2 function within this pathway. Measurement of intracellular cAMP revealed significantly reduced levels in all three mutants compared to that of WT, with the Δ*CgCFEM1/2* double mutant showing the most severe reduction (Fig. 8A). Expression analysis of cAMP-related genes indicated that the reduced cAMP in Δ*CgCFEM1* and Δ*CgCFEM1/2* correlated with downregulation of the cAMP synthesis gene *CgMac1*, whereas in Δ*CgCFEM2* and Δ*CgCFEM1/2*, it associated with upregulation of the cAMP degradation gene *CgPdeH* (Fig. 8B, C). These results demonstrate that CgCFEM1 and CgCFEM2 maintain cAMP homeostasis via distinct mechanisms-CgCFEM1 primarily promotes synthesis, while CgCFEM2 suppresses degradation.

Given the complex crosstalk between cAMP-PKA and Pmk1 MAPK signaling pathways in regulating appressorium formation [6, 28], we assessed the phosphorylation state of Pmk1 in the mutants. Western blot analysis confirmed significantly lower Pmk1 phosphorylation in all mutants relative to WT (Fig. 8D). The double mutant exhibited an intermediate phosphorylation level, consistent with the additive effect of the two genes on cAMP accumulation. These findings indicate that CgCFEM1 and CgCFEM2 converge on Pmk1 activation by maintaining cAMP levels, thereby regulating appressorium formation in *C. gloeosporioides*.

## Discussion

The successful colonization of fungal pathogens to host plants is a complex process that requires precise coordination of fungal developmental biology and the active suppression of host immunity [29–31]. During plant-pathogens interactions, pathogens deploy a number of effectors into host cells. Fungal effectors play a crucial role in modulating fungal development and regulating plant immunity [32, 33]. Among these, effectors containing the CFEM domain represent a fascinating class of key players uniquely involved in fungal pathogenicity [34–36].​ In the rubber tree anthracnose fungus, *C. gloeosporioides*, our previous work identified CgCFEM1 as a crucial virulence factor [23]. ​​​​Here, we demonstrate that its closest homolog, CgCFEM2, plays a complementary yet distinct role, and together they synergistically orchestrate the infection cycle by modulating core signaling pathways.

Our genetic analysis confirms that both are indispensable for full virulence, but they exhibit a clear synergistic relationship. The significant attenuation of virulence in the Δ*CgCFEM2* mutant, and the dramatically more severe defect observed in the double mutant Δ*CgCFEM1/2* (Fig. 3), provides compelling genetic evidence for this synergy. This synergy is rooted in their distinct molecular mechanisms for regulating cAMP homeostasis, a central finding of our study. While CgCFEM1 promotes cAMP synthesis by upregulating the adenylate cyclase gene *CgMac1*, CgCFEM2 inhibits cAMP degradation by repressing the phosphodiesterase gene *CgPdeH* (Fig. 8). This dual regulation, targeting synthesis and degradation, ensures precise control of cAMP levels, which is critical for appressorium formation and subsequent Pmk1 MAPK activation. The distinct regulatory patterns of conidiation-related genes in the single mutants further support their engagement in overlapping yet distinct developmental pathways. For instance, *CgCon6*, *CgCon10*, and *CgHox7* were downregulated in Δ*CgCFEM2* and Δ*CgCFEM1/2*, while *CgCos1* was upregulated in Δ*CgCFEM1* and ΔCgCFEM1/2 (Fig. 4E). This differential regulation is reminiscent of the sporulation gene expression changes observed in *Penicillium expansum* Δ*PeCFEM* mutants [35].

In addition to the cAMP-PKA pathway, our data implicate CgCFEM1 and CgCFEM2 in modulating TOR signaling. The mutants exhibited hallmarks of hyperactive TOR signaling: elongated germ tubes with excessive mitosis (Fig. 6B), reduced sensitivity to rapamycin, and elevated phosphorylation of the TOR downstream target p70-S6K (Fig. 7). These phenotypes are consistent with studies in *Magnaporthe oryzae*, where disruption of the MoWhi2-MoPsr1 complex leads to inappropriate TOR activation, aberrant cell cycle progression, and impaired appressorium formation [14]. The partial rescue of the appressorium formation defect by rapamycin in our mutants parallels findings in *M. oryzae* where pharmacological inhibition of TOR restores normal differentiation [27, 37, 38], supporting a model where CgCFEM1 and CgCFEM2 act as upstream regulators that restrain TOR activity, ensuring a timely cell cycle exit and proper appressorium differentiation.

While early models proposed a linear hierarchy with Pmk1 acting downstream of cAMP-PKA [6], accumulating evidence supports a more complex crosstalk between these pathways. For instance, studies in *M*. *oryzae* have shown that the Pmk1 MAPK pathway, while regulated by cAMP signaling, also functions independently in later stages of appressorium development [39]. Similarly, in *Colletotrichum orbiculare*, Harata and Kubo demonstrated that Ras GTPase activating protein CoIra1 regulates both cAMP and MAPK signaling pathways through CoRas2, indicating their interconnected nature [28]. Our findings align with this revised view, demonstrating that cAMP homeostasis maintained by CgCFEM1 and CgCFEM2 is critical for proper Pmk1 activation, but not necessarily through a simple linear relationship.

While our data establish CgCFEM1 and CgCFEM2 as key regulators of fungal development, we also acknowledge their potential role in direct host manipulation. CFEM effectors in other pathosystems, such as PstCFEM1 in *Puccinia striiformis* [36] and CfEC12 in *Colletotrichum fructicola* [29], function primarily by suppressing host immunity. We further demonstrated that CgCFEM2 possesses a functional signal peptide and can suppress chitin-induced ROS burst in rubber tree protoplasts (Figure S4), supporting its classification as a bifunctional effector involved in both developmental regulation and immune suppression—a feature increasingly recognized in phytopathogenic fungi [32, 33].

In-depth understanding of CgCFEM1/CgCFEM2-related fungal development reveals how a phytopathogenic fungi can orchestrate multiple signal pathways to modulate the development of conidia and invasive structures, achieving successful invasion of host cells. Future work will focus on identifying the direct upstream signals and downstream targets of these effectors, as well as their potential host targets, to further elucidate the molecular network governing *Colletotrichum* pathogenesis.

## Conclusions

This study establishes that CgCFEM1 and CgCFEM2 are non-redundant, synergistic pathogenicity-related effectors pivotal of *C. gloeosporioides.* They orchestrate infection by regulating conidiation and the morphogenesis of invasive structures. Mechanistically, they fine-tune the TOR pathway for appressoria development and, crucially, maintain cAMP homeostasis via distinct routes—CgCFEM1 by promoting synthesis and CgCFEM2 by inhibiting degradation. This regulation ensures activation of the downstream Pmk1-MAPK cascades. Our findings reveal a sophisticated mechanism where CFEM effector proteins modulate core signaling pathways to drive fungal pathogenesis.

## Supplementary Information

Additional file 1: Figure S1. Evolutionary analysis of CgCFEM1 and CgCFEM2. Figure S2. Construction and verification of the *CgCFEM2* knockout mutant and complementation strains. Figure S3. PCR Diagnosis for the *CgCFEM1* and *CgCFEM2* double-gene knockout mutant. Figure S4. Identification of signal peptide (SP) secretory activity of CgCFEM2 and effects of CgCFEM2 on ROS production. Figure S5. The effects of *CgCFEM1* and *CgCFEM2* deletion on the conidial germination of *C. gloeosporioides*.

Additional file 2: Table S1. Informations of CFEM effectors in *C. gloeosporioides*. Table S2. Primers used in the study.

Additional file 3. Full-length, unprocessed blots.

## Data Availability

All data generated or analyzed during this study are included in this article and its supplementary information files.

## Abbreviations

AP: Appressorium
cAMP: Cyclic adenosine monophosphate
CFEM: Common in fungal extracellular membranes
CFW: Calcofluor white
CM: Complete medium
CWI: Cell wall integrity
DAPI: 2-(4-Amidinophenyl)-6-indolecarbamidine dihydrochloride
dpi: Days post-inoculation
GT: Germ tube
H₂O₂: Hydrogen peroxide
hpi: Hours post-incubation
HPT: Hygromycin phosphotransferase gene
IH: Invasive hypha
MAC: Adenylate cyclase
MAPK: Mitogen-activated protein kinase
MM: Minimal medium
PDA: Potato dextrose agar
PDE: Phosphodiesterase
PKA: Protein kinase A
qRT-PCR: Quantitative real-time-polymerase chain reaction
Rap: Rapamycin
S6K: P70-S6 kinase
TOR: Target of rapamycin
WT: Wild type
Y2H: Yeast two-hybrid
