# Supplementary material for: CgCFEM1 and CgCFEM2 modulate virulence in Colletotrichum gloeosporioides by integrated regulation of TOR and cAMP-PKA signaling pathways
Source: BMC Microbiol. 2026 Apr 28;26:556. doi: 10.1186/s12866-026-04969-x (PMC13277146; doi:10.1186/s12866-026-04969-x)
Supplement: Supplementary file 1 — Additional file 1: Figure S1. Evolutionary analysis of CgCFEM1 and CgCFEM2. Figure S2. Construction and verification of the CgCFEM2 knockout mutant and complementation strains. Figure S3. PCR Diagnosis for the CgCFEM1 and CgCFEM2 double-gene knockout mutant. Figure S4. Identification of signal peptide (SP) secretory activity of CgCFEM2 and effects of CgCFEM2 on ROS production. Figure S5. The effects of CgCFEM1 and CgCFEM2 deletion on the conidial germination of C. gloeosporioides. [file 12866_2026_4969_MOESM1_ESM.docx]

**Figure S1. Evolutionary analysis of CgCFEM1 and CgCFEM2. (A)** Phylogenetic tree of the five CFEM effectors in *C. gloeosporioides*. The tree was constructed using the maximum likelihood method **(B)** Multiple sequence alignment of CFEM domain in CgCFEM effectors.

**Figure S2. Construction of the *CgCFEM2* knockout mutant and complementation strains. (A)** Schematic representation of the homologous recombination strategy used for gene knockout. The *Magnaporthe oryzae* acetolactate synthase gene cassette conferring resistance to chlorimuron ethyl was used as a selective marker. PCR primers, represented by black triangles, were designed to detect the integration of recombinant fragments and confirm the deletion of the target gene. WT: wide type; the symbol “Δ” indicates the gene knockout mutants. (**B)** Diagram illustrating the complementation strategy for the *CgCFEM2* knockout mutant. The full-length coding sequence of *CgCFEM2* and its native 1 kb promoter were cloned into a plasmid containing the *Aspergillus nidulans* tryptophan synthase terminator (TtrpC) and the hygromycin phosphotransferase gene (HPT) as a selectable marker. **(C)** PCR analysis confirming the correct integration of recombinant fragments at the target locus and verifying the presence of the gene. **(D)** Southern blot analysis of WT and Δ*CgCFEM2*.

**Figure S3.** **PCR Diagnosis for the *CgCFEM1 and CgCFEM2* double-gene knockout mutant.**

**
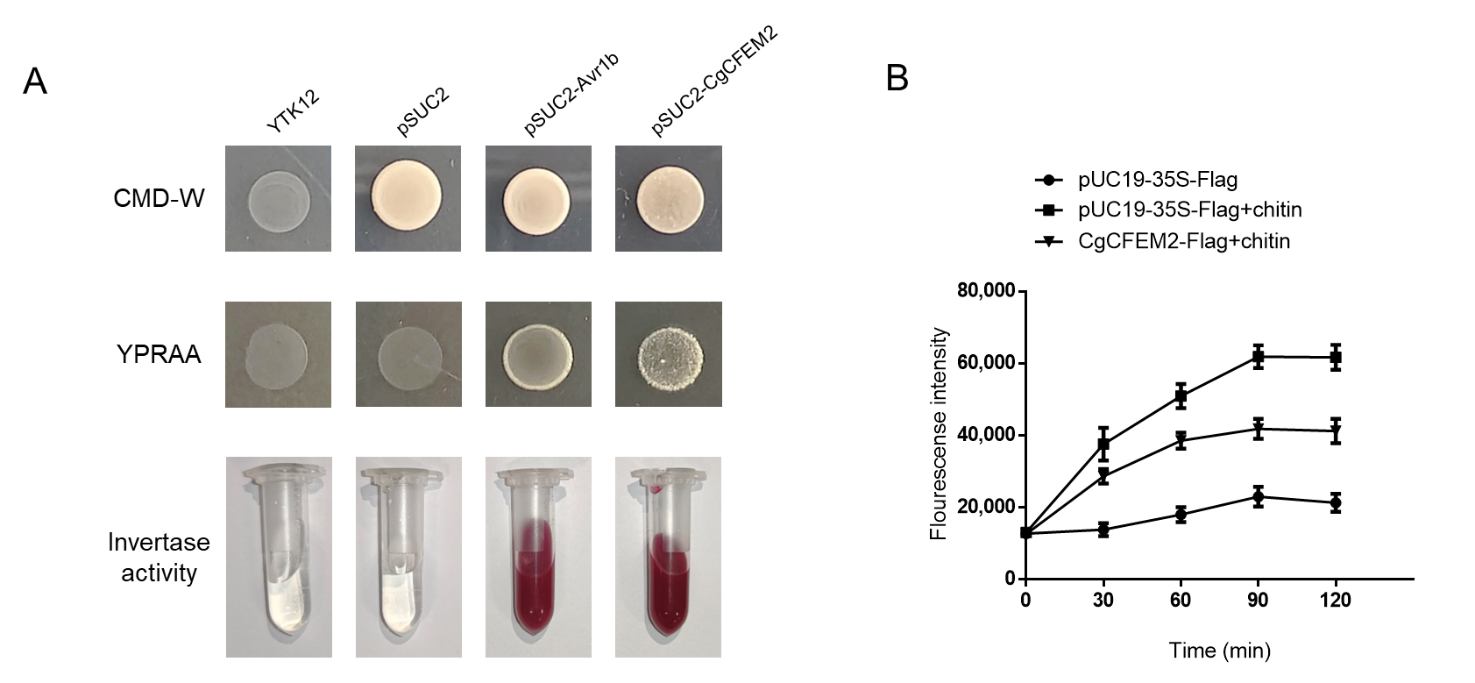
**

**Figure S4. Identification of signal peptide (SP) secretory activity of** **CgCFEM2 and effects of CgCFEM2 on ROS production. (A)** Functional validation of the SP of CgCFEM2 using the yeast invertase secretion assay. The invertase-defective yeast YTK12 carrying pSUC2-Avr1b and pSUC2- CgCFEM2 could grow on both CMD-W and YPRAA medium. YTK12 and YTK12 carrying empty vector pSUC2 were used as negative controls. Invertase activity was detected by 2,3,5-triphenyltetrazolium chloride (TTC). The red color represents the invertase activity. **(B)** Effects of CgCFEM2 on the ROS production induced by chitin. ROS productions were analyzed in rubber tree mesophyll protoplasts expressing empty vector without/with chitin treatment and expressing CgCFEM2 with chitin treatment. ROS contents were measured by DCFH2-DA.

**Figure S5.** **The effects of *CgCFEM1* and *CgCFEM2* deletion on the conidial germination of** ***C. gloeosporioides*.** **(A)** Conidial germination morphology in WT, Δ*CgCFEM1*, Δ*CgCFEM2* and Δ*CgCFEM1/2* strains in 4 h. Scale Bars = 10 µm. **(B)** Statistical analysis of conidial germination in WT, Δ*CgCFEM1*, Δ*CgCFEM2* and Δ*CgCFEM1/2* strains. Data are shown as the means ±SD from three independent experiments, and columns with different letters indicate a signiﬁcant difference (*p* < 0.05).
