# Supplementary figures and images for: CgCFEM1 and CgCFEM2 modulate virulence in Colletotrichum gloeosporioides by integrated regulation of TOR and cAMP-PKA signaling pathways
Source: BMC Microbiol. 2026 Apr 28;26:556. doi: 10.1186/s12866-026-04969-x (PMC13277146; doi:10.1186/s12866-026-04969-x)

**Supplementary Full-length blots**

**The yellow solid line outlines the border of the membrane.**


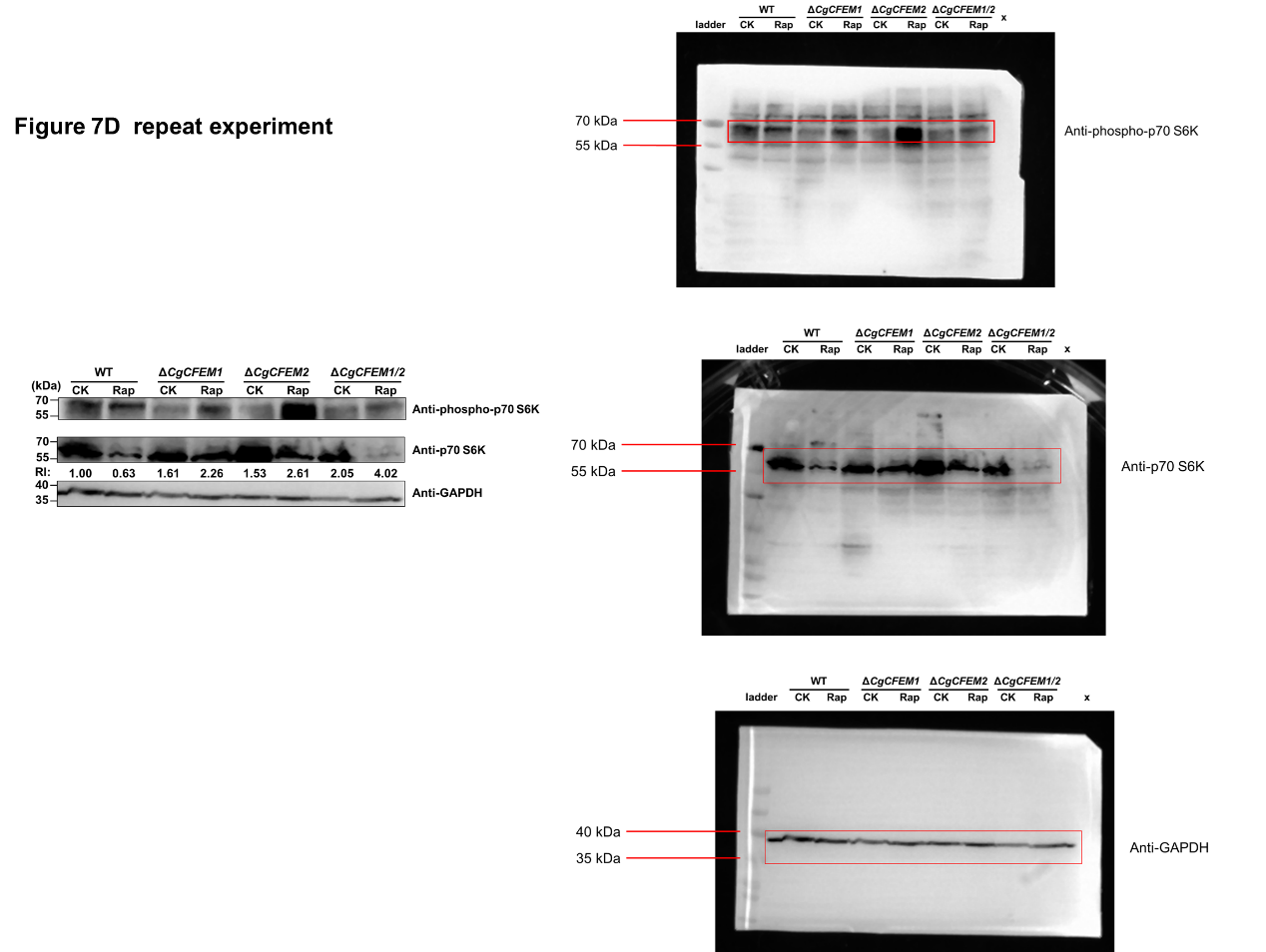

Supplement: Supplementary file 3 — Additional file 3. Full-length, unprocessed blots. [file 12866_2026_4969_MOESM3_ESM.docx]
